# Suppression of FPR2 expression inhibits inflammation in preeclampsia by improving the biological functions of trophoblast via NF-κB pathway

**DOI:** 10.1007/s10815-022-02395-2

**Published:** 2022-01-11

**Authors:** Shuxian Li, Anna Li, Liping Zhai, Yaqiong Sun, Ling Yu, Zhenya Fang, Lin Zhang, Yanjie Peng, Meihua Zhang, Xietong Wang

**Affiliations:** 1 Key Laboratory of Birth Regulation and Control Technology of National Health Commission of China, Maternal and Child Health Care Hospital of Shandong Province, 238 Jingshi East Road, Jinan, 250014 Shandong China; 2 Department of Obstetrics and Gynecology, Provincial Hospital Affiliated To Shandong University, Jinan, 250021 China; 3 Shandong Provincial Institute of Endemic Disease Control, Jinan, 250014 China

**Keywords:** Preeclampsia, FPR2, Inflammation, WRW4, NF-κB

## Abstract

**Purpose:**

The dysfunction of trophoblast during inflammation plays an important role in PE. Formyl peptide receptor 2 (FPR2) plays crucial roles in the development of inflammation-associated disease. This present study aimed to explore the effect of FPR2 on a trophoblast cellular model of preeclampsia.

**Methods:**

The expression of FPR2 in placenta was detected by immunohistochemical staining and western blotting. Transfection of siRNA was used to knockdown *FPR2* in HTR-8/SVneo cells. Inflammatory cytokines were detected by ELISA. CCK8, Transwell, wound healing, FACS and tube formation assays were performed to observe the abilities of cell proliferation, migration, invasion, apoptosis and angiogenesis. Western blotting was implemented to clarify that NF-κB signaling pathway was downstream of FPR2.

**Results:**

The expression levels of FPR2 were higher in placental tissues of patients with PE. Knockdown of *FPR2* expression by si*FPR2* or inhibition of its activity by WRW4 decreased the release of proinflammatory cytokines in HTR8/SVneo cells treated with LPS. Knockdown of *FPR2* expression or inhibition of its activity further reversed the LPS-induced attenuation of the proliferation, migration, invasion and angiogenesis and increase in apoptosis in HTR8/SVneo cells. Moreover, the NF-κB signaling pathway was activated in both placental tissues of patients with PE and LPS-treated HTR8/SVneo cells. However, the activation was attenuated when *FPR2* was knocked down or inhibited.

**Conclusion:**

Suppression of FPR2 expression alleviated the effects of inflammation induced by LPS on trophoblasts via the NF-κB signaling pathway, which provided a novel and potential strategy for the treatment of PE.

## Introduction

Preeclampsia (PE) is a pregnancy disorder characterized by high blood pressure, proteinuria and end-organ dysfunction at a gestational age of ≥ 20 weeks [1]. It is one of the leading causes of maternal and neonatal morbidity and mortality worldwide [2]. Although the pathophysiological mechanism of PE remains ambiguous, accumulating studies have indicated that excessive inflammation influences the development and severity of PE [3–5]. Mounting evidence has shown that abnormal trophoblast differentiation and invasion are associated with an imbalanced maternal immune response [6]. Excessive release of inflammatory cytokines can trigger inadequate trophoblast invasion and impaired spiral arteries remodeling [7]. It is reasonable to assume that excessive inflammation is one of the causes of PE [8]. At present, inhibition of inflammation is considered a promising therapeutic strategy to prevent the progression of PE [9]. However, despite various hypotheses regarding the association between inflammation and PE, its exact mechanism of action remains unclear.

Formyl peptide receptor 2 (FPR2) belongs to the family of G protein-coupled receptor (GPCRs) and plays an important role in inflammation by interacting with multiple ligands [10]. In humans, FPR2 (or FPRL1/ALX) is expressed in a variety of cells, including lymphocytes, immune, neuronal and epithelial cells, as well as in different tissues, including the lung, kidney and placenta [11, 12]. FPR2 is known to be a pattern recognition receptor that can detect bacteria and microorganisms through formylated peptides and in turn, trigger proinflammatory reactions [13, 14]. By contrast, FPR2 has also been shown to exert inhibitory effects on inflammation and can restore homeostasis when activated by anti-inflammatory lipid mediators, such as lipotinA4 (LXA4), resolvin D1 (RVD1) or the glucocorticoid-modulated protein annexin A1 [15–17]. In summary, these studies have shown that FPR2 is a vital inflammation-related receptor and therefore may be considered as a novel target for the treatment of PE.

The NF-κB signaling pathway plays a prominent role in inflammation by regulating the expression of proinflammatory factors, such as tumor necrosis factor-α (TNF-α), interleukin (IL)-1β and IL-6 [18]. Simultaneously, it has been reported that NF-κB signaling pathway regulates cell proliferation and apoptosis [19, 20]. FPR2 has been reported to affect cell apoptosis and inflammation via the NF-κB signaling pathway [21]. The current study aimed to further investigate the effects of FPR2 on a lipopolysaccharide (LPS)-induced trophoblastic inflammation model by examining the contribution of the NF-κB signaling pathway.

In the present study, we explored the role of FPR2 in the progression of PE. The increased expression of FPR2 in placental samples with PE indicated that FPR2 had an effect on PE. Suppression of FPR2 not only reduced the increase in proinflammatory factors caused by LPS, but also reversed the effects of LPS on proliferation, apoptosis, invasion, migration and angiogenesis of trophoblasts. Meanwhile, the expression of NF-κB signaling pathway was closely related to the suppression of FPR2. Therefore, FPR2 could be provided as a therapeutic interventional target to prevent PE.

## Materials and methods

### Patient samples collection

The clinical specimens were obtained from The Maternal and Child Healthcare Hospital of Shandong Province. The study protocol conformed to the relevant ethical regulations of clinical specimen research. All patients signed the relevant informed consent forms in order to participate in the study. The research protocol was approved by the Ethics Committee of Maternal and Child Healthcare Hospital of Shandong Province (NSFC: NO.2021–036). Placental samples were selected from 10 patients with severe PE (25–33 years old) who exhibited signs of hypertension and proteinuria (systolic blood pressure (SBP) ≥ 160 mmHg, diastolic blood pressures ≥ 110 mmHg, proteinuria > 300 mg /day). Placental tissues obtained from healthy pregnant women aged 25–33 were used as the control. The placental tissues were immediately collected from the maternal side of the placenta and the tissues with hemorrhage, infarction or calcification were avoided to select. The collected tissues were rinsed with PBS to remove residual blood. One part of the tissue was stored in liquid nitrogen at -80 °C, and the remaining parts were fixed with 4% paraformaldehyde, embedded in paraffin and sectioned.

### Cell line and culture

The human trophoblast cell line HTR8/SVneo, originating from human placental trophoblast cells, was purchased from The American Type Culture Collection (ATCC, USA). HTR8/SVneo cells were cultured on cell plate (3736, Corning, Inc., USA) with RPMI-1640 medium (Invitrogen; Thermo Fisher Scientific, Inc., USA) containing 10% fetal bovine serum (Invitrogen; Thermo Fisher Scientific, Inc., USA), 1% sodium pyruvate (Invitrogen; Thermo Fisher Scientific, Inc., USA) and 1% penicillin and streptomycin (Invitrogen; Thermo Fisher Scientific, Inc., USA). The cells were incubated at 37 °C in 5% CO_2_ and allowed to grow to 80% confluence. Subsequently, they were seeded (3 × 10^5^ cells/well, respectively) into 6-well plates and treated with LPS (100 ng/ml; Sigma-Aldrich; Merck KGaA, USA) for 12 h to induce a trophoblastic inflammatory response. The cells were treated with FPR2 antagonist WRW4 (10 μg/ml; Tocris Bioscience, UK) for 30 min prior to LPS addition [16]. The samples from the cells and the supernatant were collected for subsequent experiments.

### Histology and immunochemistry

The placental tissues were embedded in paraffin and sectioned. The slices were boiled with 0.01 mol/l citrate buffer (pH 6.0) for antigen retrieval and cooled to room temperature. The activity of endogenous peroxidase in tissues was removed using methanol containing 3% H_2_O_2_, and the cells were permeabilized with 0.1% Triton X-100 for 30 min prior to blocking in 10% goat serum (Solarbio Science & Technology Co., Ltd., China) for an additional 30 min. The slices were incubated with a primary antibody against FPR2 (1:100, ab63022, Abcam USA) overnight at 4 °C and subsequently incubated with the corresponding secondary antibody (G1213-100UL, Servicebio Technology Co., Ltd., China) at 37 °C for 60 min. Finally, they were washed with PBS and stained with diaminobenzidine tetrahydrochloride (Solarbio Science & Technology Co., Ltd., China).

### Cell transfection with siRNA Oligonucleotides

*FPR2* siRNA (EHU017941, Sigma-Aldrich; Merck KGaA, USA) was used to knockdown the endogenous gene, *FPR2*, in HTR8/SVneo cells. The cells were seeded at a density of 3 × 10^5^/well into 6-well plates and cultured to 50% confluence. Complete medium was replaced with Opti-MEM™ (Invitrogen; Thermo Fisher Scientific, Inc.), and the cells were then transfected using 2 μL/mL Lipofectamine® 2000 transfection reagent (Invitrogen; Thermo Fisher Scientific, USA) and 600 ng/mL siRNA according to the manufacturer’s protocol. Following 8 h of incubation, the medium was changed to complete medium, and the cells were cultured for 48 h. Subsequently, the cells were harvested for western blotting, enzyme-linked immunosorbent assay (ELISA), and proliferation, apoptosis, Transwell, wound healing and tube formation assays.

### Enzyme-linked Immunosorbent Assay (ELISA)

The concentration levels of IL-1β, IL-6, IL-10 and TNF-α in the cell culture supernatants were measured using ELISA kits (EK0392, EK0410, EK0416, EK0525, Boster Biological Technology, China) according to the manufacturers’ protocols.

### Western Blotting

Placental tissues were homogenized using Potter–Elvehjem Tissue Grinders on ice and dissolved in RIPA (radio immunoprecipitation assay) lysis buffer (Beyotime Institute of Biotechnology, China) containing 1 mmol/L PMSF (phenylmethanesulfonyl fluoride) (Beyotime Institute of Biotechnology, China). The BCA (bicinchoninic acid) assay was performed to quantify the concentration of the extracted protein. The protein samples (20–30 μg) were separated via SDS-PAGE and transferred to polyvinylidene membranes (MilliporeSigma, USA). Following blocking of the membranes with 5% non-fat milk in Tris-buffer saline containing 0.05% Tween 20 (TBST), the membranes were incubated with primary antibodies (anti-FPR2, 1:1,000, ab63022, Abcam, USA; anti-IκBα, 1:1,000, 4814, Cell Signaling Technology, Inc., USA; anti-NF-κB (p65), 1:1,000; GB11997, anti-phosphorylated (p)-NF-κB (p65), 1:1000, GB11142-1; anti-β-actin, 1:1,000; GB15001, all Wuhan Servicebio Technology Co., Ltd., China) overnight at 4 °C. The membranes were subsequently incubated with an HRP-conjugated secondary antibody (1:3,000, G1213-100UL, Servicebio Technology Co., Ltd., China) for 1 h at 37 °C. The protein signals were visualized using the ECL system. The protein bands were acquired and semi-quantified using an Amersham Imager 600 (Amersham; Cytiva, USA) and analyzed using ImageJ (National Institutes of Health, Bethesda, USA) software.

### Cell Counting Kit-8 (CCK8) assay

HTR8/SVneo cells (3 × 10^3^ cells/200 µl) were seeded into 96-well plates. Following 12 h of incubation, the cells were treated with LPS (100 ng/ml; Sigma-Aldrich; Merck KGaA, USA) for 12 h to elicit a trophoblast inflammatory response. The cells treated with WRW4 (10 μg/ml; Tocris Bioscience, UK) were treated for 30 min prior to induction with LPS. After 24 h, the cells were treated with CCK-8 (10 μl/well; Sigma-Aldrich; Merck KGaA, USA) for 1 h. Finally, the absorbance was measured at 450 nm with a microplate reader (Norgen Biotek Corp., Canada).

### Apoptosis assay

The induction of apoptosis was detected by an Annexin V-FITC/PI Apoptosis Detection kit (BD Biosciences, USA). HTR8/SVneo cells were seeded into 6-well plates at the density of 4 × 10^4^ cells/well for 12 h. Subsequently, the cells were treated with LPS (100 ng/ml; Sigma-Aldrich; Merck KGaA, USA) for 12 h. The cells were treated with WRW4 prior to treatment with LPS, harvested and washed with PBS. Following staining with Annexin V-FITC and PI for 20 min in light-resistant conditions, the cells were analyzed using a FACSCalibur flow cytometer (BD Biosciences, USA).

### Wound-healing assay

HTR8/SVneo cells were seeded at the density of 6 × 10^4^ cells/100 µl into a Culture-Insert 4 Well (Ibidi GmbH, Germany). Following culture for 12 h, the culture inserts were gently removed using a sterile tweezer. Following washing three times with PBS in order to remove cell debris, the cells were cultured in fresh medium. Wound healing was observed at 0 and 24 h using a microscope (4X objective). The wound area was calculated using ImageJ (National Institutes of Health, Bethesda, USA) software.

### Invasion assay

The 24-well plate was equipped with polycarbonate membrane Transwell inserts containing 8-µm pores (Corning, Inc., USA). The bottom membrane of the plate was covered with 50 µL (1 mg/mL) Matrigel matrix (Becton, Dickinson and Company, USA) for invasion. Following incubation for 2 h at 37 °C, the Matrigel was solidified and HTR8/SVneo cells (2 × 10^5^ cells in 200 µL serum-free medium) were seeded in the upper chamber. A total of 600 µL medium with 10% FBS was added to the bottom chamber. Cell invasion was allowed to occur for 12 or 24 h, and then the culture medium in the chamber was removed. Following washing with PBS, the cells in the upper chamber were removed with a cotton swab, and 4% paraformaldehyde was added into each chamber to fix the cells remaining in the bottom of the chamber for 20 min. Crystal violet (1%) was added in the chamber to stain the cells for 20 min. The images were obtained using an inverted microscope (Olympus Corporation, Japan), and the number of invading cells was counted in five randomly selected fields for each chamber.

### Tube formation assay

HTR8/SVneo cells were seeded at the density of 8 × 10^4^ cells/100 µl into Matrigel-coated 96-well plates, and then different treatments (100 ng/ml LPS or 10 µg/ml WRW4) were performed; LPS and WRW4 were used in order to induce tube formation. Following 4 h of incubation, tube formation was observed using an inverted microscope in at least five randomly selected fields of view. The tube length per field was calculated using ImageJ (National Institutes of Health, Bethesda, USA) software.

### Statistical analyses

All experiments were performed at least three times. All data were analyzed using GraphPad Prism version 8.0 (GraphPad Software, Inc., USA). The images were calculated using ImageJ (National Institutes of Health, Bethesda, USA) software. The replicate data are presented as the mean ± SEM. The differences between two or more groups were compared using an unpaired two-tailed Student’s *t* test or an ANOVA, respectively. A *p* < 0.05 was considered to indicate a statistically significant difference.

## Results

### The expression levels of FPR2 are significantly increased in placental tissues from patients with PE and HTR8/SVneo trophoblast cells treated with LPS

**Fig. 1:**
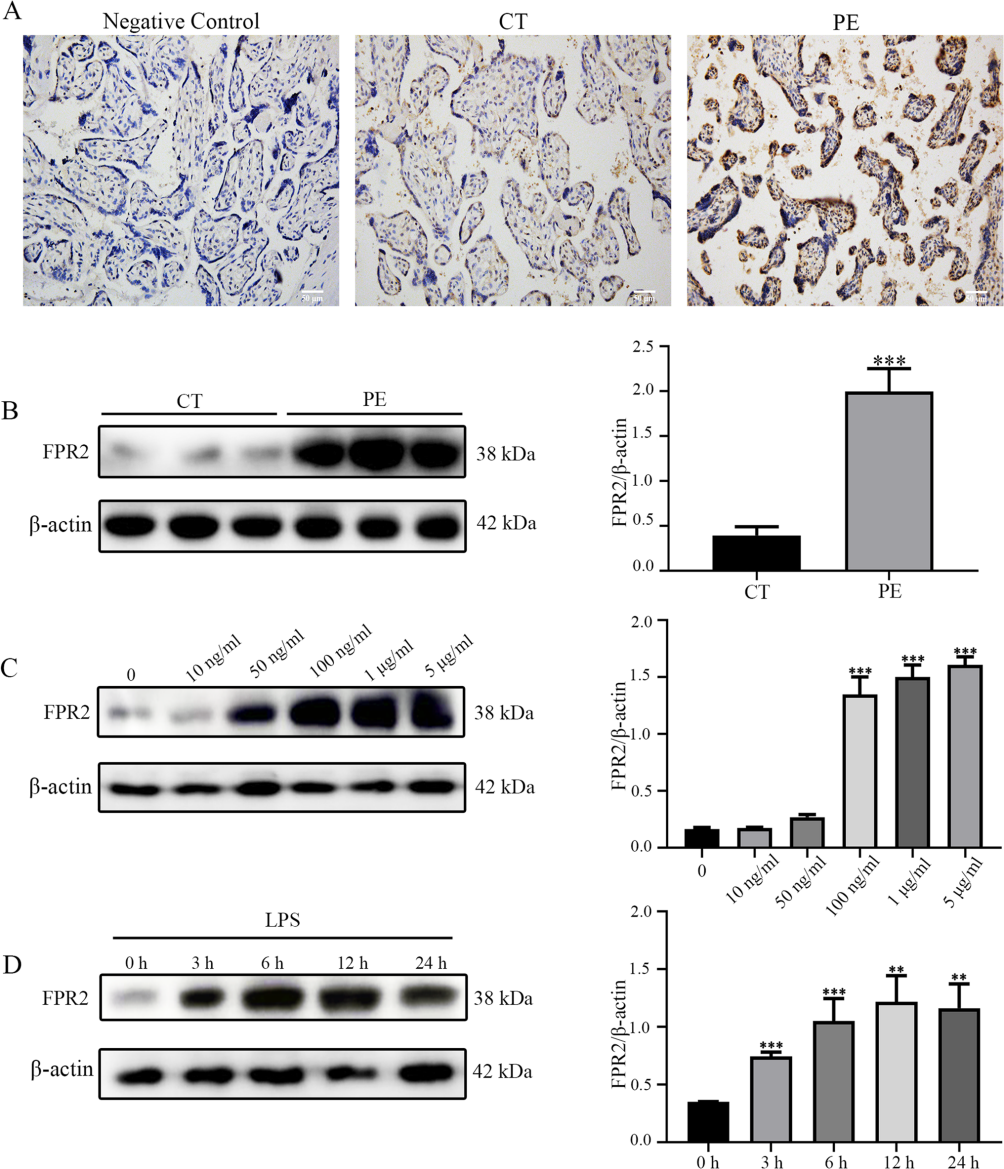
The expression levels of FPR2 were significantly increased in the placenta tissues of patients with PE. (**A**) The expression levels of FPR2 in placenta tissues from CT (n = 10) and patients with PE (n = 10) were detected using immunohistochemistry analysis. Scale bar, 50 μm. (**B**) The expression levels of FPR2 were determined using western blotting analysis (***P < 0.001: PE *vs* CON group). (**C**) The expression levels of FPR2 in response to treatment of HTR8/SVneo cells with different concentrations of LPS (0, 10, 50 and 100 ng/mL, 1 and 5 μg/mL) (***P < 0.001: LPS *vs* CON group). (**D**) The expression levels of FPR2 following different durations of treatment (0, 3, 6, 12 and 24 h) in the presence of 100 ng/ml LPS (**P < 0.01, ***P < 0.001: LPS *vs* CON group)

To explore the role of FPR2 in PE, its expression levels were examined in the placental tissues obtained from women with or without PE (Table 1). The location and expression of FPR2 in the placenta were assessed using immunohistochemical analysis (Fig. 1A). The results indicated that FPR2 was mainly expressed in trophoblasts in the placenta. The expression levels of FPR2 in PE placental tissues were considerably higher than those noted in the control group. Western blotting (Fig. 1B) indicated the same trend with that noted using immunohistochemical staining, which suggested that upregulation of FPR2 expression may be associated with PE. Moreover, the expression levels of FPR2 were detected in HTR8/SVneo cells in response to LPS, which was used to simulate the inflammatory environment noted in patients with PE. The data indicated that the expression levels of FPR2 continued to increase in a dose-dependent manner following LPS stimulation (Fig. 1C). When the concentration of LPS was 100 ng/ml, the expression levels of FPR2 were significantly increased and therefore, this concentration (100 ng/ml) was used as the final concentration of LPS. The expression levels of FPR2 were significantly increased following 3 h of LPS treatment, and no significant trends were noted with regard to the changes noted over time (Fig. 1D).

**Table 1:** Demographic and clinical characteristics of groups

Patients characteristics	Normotensive Pregnant (n = 10)	Preeclampsia (n = 10)	*p*-value
Age (year)	30 ± 5	30.1 ± 3.1	0.9343
Gestational age (week)	39.8 ± 0.3	33.96 ± 1.96	< 0.0001
Body mass index	28.82 ± 1.92	28.75 ± 4.45	0.9374
Proteinuria (mg/day)	0.052 ± 0.098	476.5 ± 96.5	< 0.0001
Systolic blood pressure (mm/Hg)	123.7 ± 10.3	172.8 ± 3.8	< 0.0001
Diastolic blood pressure (mm/Hg)	83 ± 7	115.9 ± 5.9	< 0.0001
Birth weight (g)	3330.72 ± 362.02	2516.1 ± 346.4	0.0002
Smoking status	2	1	0.5560
Abnormal fetus	No	No	-
Fetal growth restriction	No	No	-

### Inhibition of FPR2 reduces the release of proinflammatory cytokines induced by LPS in HTR8/SVneo cells

**Fig. 2:**
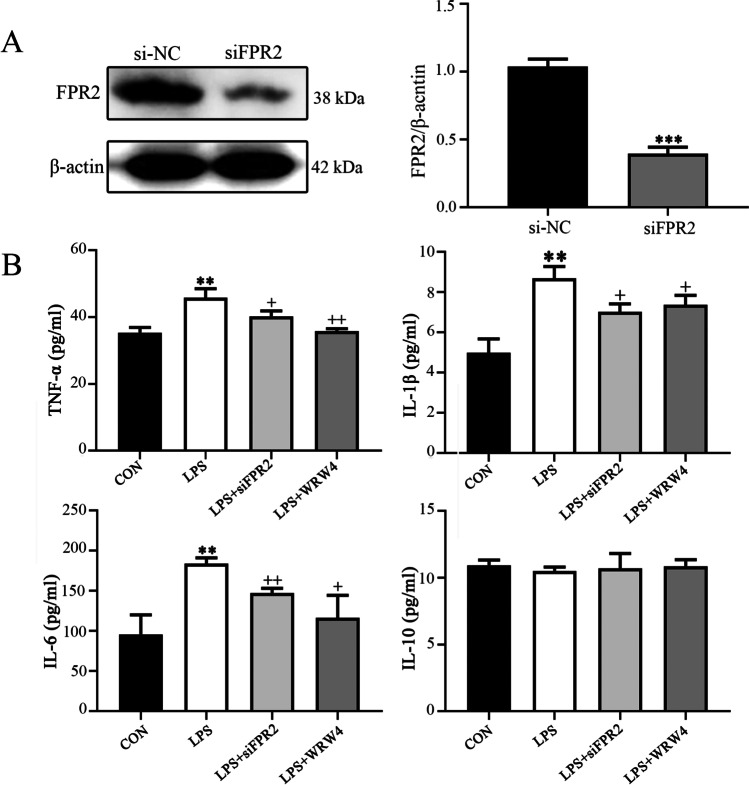
Knockdown of FPR2 expression and inhibition of its activity affects the release of inflammatory cytokines in HTR8/SVneo cells. (**A**) Determination of FPR2 protein levels in HTR8/SVneo cells transfected with siFPR2 (***P < 0.001: siFPR2 *vs* siNC group). (**B**) Estimation of the levels of proinflammatory cytokines (TNF-α, IL-1β, IL-6) and of the anti-inflammatory cytokine IL-10 in HTR8/SVneo cells. The LPS concentration used was 100 ng/ml, whereas the siFPR2 concentration used was 600 ng/ml and the WRW4 concentration was 10 μg/ml (**P < 0.01: LPS *vs* CON group; + *P* < 0.05, +  + *P* < 0.01: LPS + siFPR2 or LPS + WRW4 *vs* LPS group)

To explore the role of FPR2 in inflammation, its expression was knocked down in HTR8/SVneo cells following transfection of the cells with si*FPR2* and si-negative control. The transfection efficacy was confirmed using western blotting analysis (Fig. 2A). ELISA indicated that the expression levels of the proinflammatory cytokines, such as TNF-α, IL-1β and IL-6, were increased, while those of the anti-inflammatory cytokine IL-10 exhibited no significant changes following treatment of HTR8/SVneo cells with LPS. The changes noted in the expression levels of the proinflammatory cytokines following treatment of HTR8/SVneo cells with LPS were reversed due to the knockdown of *FPR2* expression or addition of the FPR2 antagonist WRW4 to the medium in advance (Fig. 2B). However, the expression levels of the anti-inflammatory factors remained unaltered (Fig. 2B).

### Knockdown of FPR2 expression or inhibition of its activity alleviates functional impairment of HTR8/SVneo cells induced by LPS

**Fig. 3:**
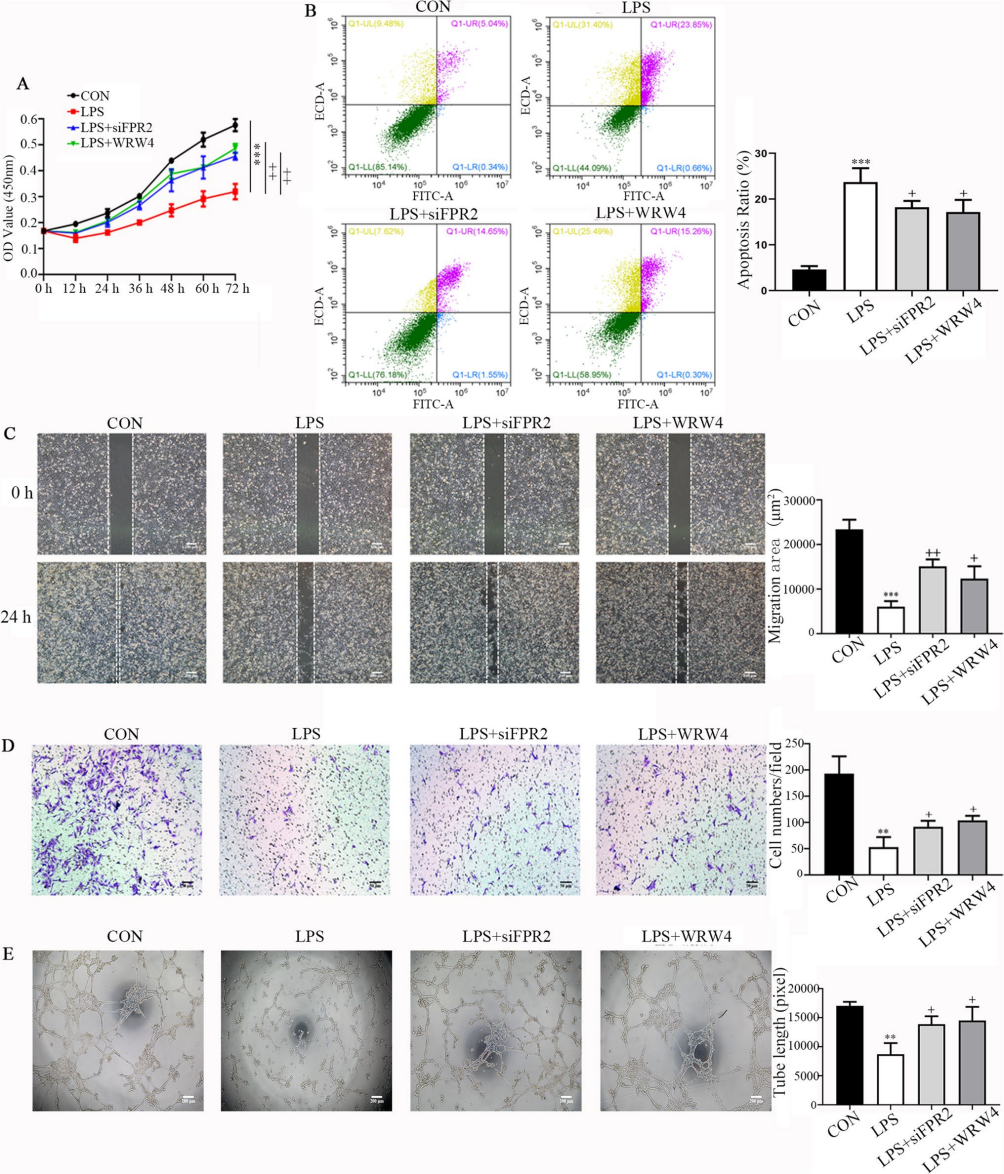
Knockdown of FPR2 expression or inhibition of its activity rescues the proliferation, apoptosis, migration, invasion and tube formation abilities of HTR8/SVneo cells pretreated with LPS. (**A**) The proliferation curves were determined using the CCK8 assay. The plots represent the proliferative ability of HTR8/SVneo cells (***P < 0.001: LPS *vs* CON group; +  + *P* < 0.01: LPS + siFPR2 or LPS + WRW4 *vs* LPS group). (**B**) Annexin V-FITC/PI staining and flow cytometry assays were used to determine the percentage of apoptotic cells following their treatment with LPS, LPS + siFPR2 and LPS + WRW4. Histogram analysis indicates the apoptotic rate of the cells in each group (***P < 0.001: LPS *vs* CON group; + *P* < 0.05: LPS + siFPR2 or LPS + WRW4 *vs* LPS group). (**C**) Representative microphotographs of the wound healing assay of HTR8/SVneo cells under different conditions at the 0 and 24 h time points. The histogram indicates the migration area (μm^2^) in each group. Scale bar, 200 μm (***P < 0.001: LPS *vs* CON group; +  + *P* < 0.01, + *P* < 0.05: LPS + siFPR2 or LPS + WRW4 *vs* LPS group). (**D**) The representative images of the Transwell invasion assay at 24 h in the different treatment groups. The histogram indicates the comparison of the cell counts in each group. Scale bar, 50 μm (**P < 0.01: LPS *vs* CON group; + *P* < 0.05: LPS + siFPR2 or LPS + WRW4 *vs* LPS group). (**E**) Representative images indicating the cell tube formation under different conditions at 4 h. Histogram indicating the quantification of the tube formation activity of HTR8/SVneo cells. Scale bar, 200 μm. (**A**-**E**) The LPS concentration used was 100 ng/ml. The siFPR2 and WRW4 concentrations used were 600 ng/ml and 10 μg/ml, respectively (**P < 0.01: LPS *vs* CON group; + *P* < 0.05: LPS + siFPR2 or LPS + WRW4 *vs* LPS group)

Patients with PE have an overactive inflammatory response. The inflammatory responses at the utero-placental interface were also found to be closely related to the dysfunction of the trophoblast. To investigate the effects of FPR2 on trophoblast inflammation, a series of assays were used to analyze proliferation, apoptosis, migration, invasion and angiogenesis. The CCK-8 assay was performed to detect the proliferative ability of HTR8/SVneo cells (Fig. 3A). The results indicated that the cell proliferative rate was significantly decreased in the presence of LPS. However, these effects were restored when *FPR2* was knocked down or inhibited by WRW4. The Annexin V/PI double staining kit was used to detect induction of HTR8/SVneo cell apoptosis. The percentage of the apoptotic cell population is shown in Fig. 3B. The apoptotic rate in the control group was 5.38%, whereas following LPS treatment of the cells, it was markedly increased to 24.51%. Knockdown of *FPR2* expression or addition of WRW4 to the cells reduced the apoptotic rate to 16.2 and 15.56%, respectively. The wound healing assay was carried out to characterize the migration of HTR8/SVneo cells. The results indicated that the cells migrated more slowly in the LPS-treated group compared with the corresponding migratory rate noted in the control group. However, following knockdown of *FPR2* expression or addition of WRW4 prior to LPS stimulation, the cell migratory rate was significantly increased (Fig. 3C). The results of the Transwell invasion experiments were similar (Fig. 3D); the invasive activity of the LPS-treated HTR8/SVneo cells was markedly decreased. These effects were notably alleviated when *FPR2* was knocked down or following the addition of WRW4 to the cells, prior to their treatment with LPS. The results of the tube formation assay indicated that FPR2 could reduce the angiogenic effects caused to HTR8/SVneo cells by LPS. The results indicated a significant decrease (approximately 50%) in the total tube length in response to LPS treatment (Fig. 3E). The formation of the tube length was improved following knockdown of *FPR2* expression or incubation of the cells with WRW4.

### FPR2 mediates inflammation via the NF-κB signaling pathway

**Fig. 4:**
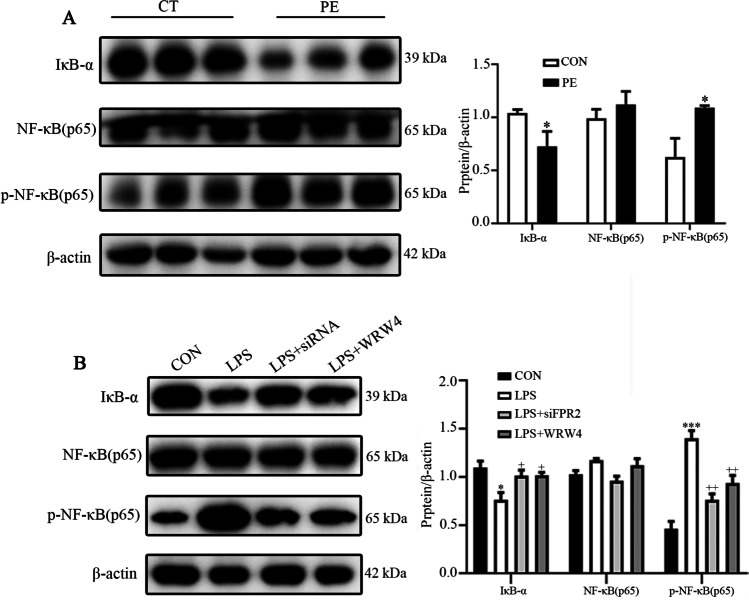
The effects of FPR2 on the activation of the NF-κB signaling pathway. (**A**) The expression levels of the IκB-α, NF-κB/p65 and p-NF-κB/p65 proteins in the placental tissues from CT and patients with PE (*P < 0.05: PE *vs* CON group). (**B**) The expression levels of IκB-α, NF-κB (p65) and p-NF-κB/p65 in HTR8/SVneo cells following their exposure to different experimental conditions. (***P < 0.001: LPS *vs* CON group; + *P* < 0.05, +  + *P* < 0.01: LPS + siFPR2 or LPS + WRW4 *vs* LPS group)

A considerable number of studies have reported the crucial role of the NF-κB signaling pathways in regulating inflammation during the progression of PE. Therefore, the present study hypothesized that FPR2 participated in the process of PE via activation of the NF-κB signaling pathway. The expression levels of IκBα, NF-κB/p65 and p-NF-κB/p65 were detected in placental tissues from patients with or without PE. The results indicated that the expression levels of IκBα in placental tissues from patients with PE were decreased compared with those of the control subjects, while the expression levels of p-NF-κB/p65 were significantly increased in placental tissues from patients with PE (Fig. 4A). The expression levels of IκBα, NF-κB/p65 and p-NF-κB/p65 in LPS-treated HTR8/SVneo cells indicated the same trend as that noted in the placental tissues compared with their corresponding controls (Fig. 4B). The results indicated that the NF-κB signaling pathway was activated in the PE and trophoblast inflammation models, while when FPR2 was knocked down or inhibited by the FPR2 specific antagonist WRW4, the activation of the NF-κB pathway was reversed.

## Discussion

Numerous studies have indicated that excessive local inflammation at the maternal–fetal interface is an important characteristic of PE [3, 22]. The discovery and application of anti-inflammatory medicines might provide novel strategies for the treatment of PE. FPR2 has been shown to be closely related to various diseases caused by inflammation [23–25]. However, the function of FPR2 in PE is not yet clear.

FPR2 participates in a variety of inflammatory responses in human by binding several kinds of ligands. It has been reported that FPR2 exhibits either proinflammatory or anti-inflammatory functions by combining with different types of ligands. Regarding its proinflammatory effect, FPR2 increases monocyte and neutrophil chemotaxis and recruitment and plays critical roles in high fat diet (HFD)-induced obesity and its associated complications by modulating inflammation mediated by macrophage M1 polarization [26, 27]. In contrast to these findings, FPR2 can inhibit and resolve inflammation following its activation by anti-inflammatory lipid mediators, such as LXA4, RVD1 or the glucocorticoid-modulated protein annexin A [28–30]. Our previous study indicated that RvD1 alleviated the inflammation of trophoblasts in vivo and in vitro by combining with FPR2 in chorioamnionitis [16]. Concomitantly, deletion of Fpr2 alleviated sepsis in LPS-treated mice and improved cardiac dysfunction, which was related to inhibition of inflammation [14]. FPR2 deletion significantly reduced the inflammatory response in lung tissues induced by LPS, which was confirmed by the decrease in the levels of specific proinflammatory factors [25]. In our present study, knockdown of *FPR2* or inhibition of its activity markedly reduced LPS-induced inflammation in human extravillous trophoblast cell line HTR8/SVneo, which was supported by the decreased proinflammatory factors, such as TNF-α, IL-1β and IL-6. The results indicated that FPR2 exhibited a proinflammatory effect in trophoblastic inflammation induced by LPS. Meanwhile, *FPR2* knockdown or inhibition of its activity reversed the ability of proliferation, migration, invasion, tube formation and apoptosis of HTR8/SVneo cells pretreated with LPS, which suggested that inhibition of FPR2 might be a potential treatment for damage of trophoblast function. In PE, due to the presence of a variety of substances or factors that cause inflammation, we speculate that FPR2 exhibits a proinflammatory effect in the early stage of the disease, while the production of anti-inflammatory mediators, such as LXA4, may be increased at later stages during resolution of inflammation. Certainly, our hypothesis requires confirmation by additional studies.

In recent years, more and more attention has been paid to the research of FPR2 in obstetrics. It was reported that FPR2 played an important role in fetal growth restriction (FGR). Reduced FPR2 contributed to trophoblast dysfunction and subsequent abnormal spiral arteriole remodeling associated with pregnancies [31]. Besides, the decreased FPR2 in human umbilical vein endothelial cells (HUVEC) led to an increasement in membrane permeability potential suggesting FPR2 might contribute to the pathophysiological mechanisms associated with placental dysfunction in FGR [32]. FPR2 increased significantly in gestational diabetes mellitus (GDM) and might be used as a marker for the prevention, clinical treatment and drug design of GDM [33]. The abnormal expression of FPR2 in a variety of obstetric diseases implies that FPR2 is closely related to obstetric disease which may provide important insights for our study. However, a limited number of studies have been performed on FPR2 in obstetric diseases related to inflammation, such as PE, recurrent spontaneous abortion and preterm birth. Moreover, the role of FPR2 in PE is still controversial. Wei et al*.* found that there was a significant increase in FPR2 expression in placenta obtained from women in the severe PE group compared with the control group in both mRNA and protein [34], while Zhang et al*.* found that the FPR2 decreased in women with PE [35]. In our present study, we found that FPR2 was increased significantly in placenta tissues from severe PE patients compared with control group using western blotting and immunohistochemical analysis. We had also proved that suppression of FPR2 alleviated the effect of inflammation induced by LPS on trophoblasts in vitro*.* Our results showed that FPR2 was involved in the local inflammatory reaction of placenta in patients with PE, which might aggravate the damage of PE. The different results obtained by researchers may be related to the severity of disease and the heterogeneity of clinical samples. The effect and mechanism of FPR2 in PE need to be further clarified and discussed.

In addition, we explored the signaling pathways associated with FPR2 in regulation of trophoblast function under an inflammatory state. LPS can interact with toll-like receptor (TLR)-4 on cell membrane and triggers a cascade of signaling events associated with a plethora of pathophysiological responses [36]. Many studies have shown that LPS can activate TLR-4 in a variety of trophoblast cell lines such as HTR8/SVneo, JEG3 and BeWo and triggers further activate downstream signaling pathways such as NF-κB, MAPK and STAT, promoting the occurrence and expansion of inflammatory reactions [7, 37]. The study was focused on the interaction of FPR2 with the NF-κB signaling pathway, which is one of the classical signaling pathways involved in the regulation of inflammation and has been shown to participate in the development of PE [38, 39]. In our present study, IκB-α was decreased, while p-NF-κB/p65 was increased significantly in placental tissues derived from patients with PE, as well as in the LPS-treated HTR8/SVneo cell line, suggesting the NF-κB signaling pathway was activated in placenta in PE patients and in HTR8/SVneo cell line treated with LPS. It was also shown that the inflammatory response was reduced by knockdown of *FPR2* expression or inhibition of its activity, which was also attributed to the blockage of the NF-κB signaling pathway, as shown by the reversal in the expression levels of IκB-α and p-NF-κB/p65. The results suggested that FPR2 played a role in inflammation via the NF-κB signaling pathway. Moreover, some literature indicated that FPR2 might affect inflammation-related signal pathways through downstream genes such as PPARγ or Nrf2 [25, 40]. In our previous research, our team found that PPARγ signaling was involved in NF-κB signaling pathway [16], but we did not discover the Nrf2 changes in in our present study. The exact mechanism of FPR2 involved in PE needs to be further explored.

Besides FPR2, FPR1 (formyl peptide receptor 1), which is another important member of the G-protein coupled pattern recognition receptor family, is also associated with inflammation induced by LPS. It is reported that LPS induces the elevation in FPR1 mRNA levels by promoting both enhanced transcription and mRNA stability and this effect can be mediated by the intermediate production of secreted stimuli to enhance the expression of FPR1 [41, 42]. Downregulation of FPR1 abated LPS-induced inflammatory injury and apoptosis by upregulating the activity of MAPK signaling pathway in murine chondrogenic ATDC5 cells [42]. Regarding the role of FPR1 in trophoblasts, it was found that FPR1 affected the implantation of BeWo trophoblast cells into uterine epithelial cells by acting with AnxA1, which mimicked the embryo implantation in vitro [43]. The research of FPR1 has greatly inspired us to further explore the related functions of FPR2. The underlying mechanism of FPR1 involved in FPR2 contributes to further understanding about the pathogenesis of PE which is a valuable direction for our continued exploration.

The current study exhibits certain limitations, such as the lack of an inflammatory model that clarify the mechanism of the Fpr2-related dysfunction of trophoblasts in vivo and replication in primary trophoblast cells or different trophoblast cell lines. In addition, the genetic expression analysis of FPR2 and its molecular mechanism in other embryonic development-related primary cells (such as HUVEC) are essential to clarify its role in the pathogenesis of PE. These limitations should be further investigated in future.

In conclusion, our study provided evidence to suggest that FPR2 might play an important role in the etiology and pathogenesis of PE. FPR2 inhibition relieved inflammation by affecting the NF-κB signaling pathway. It is important to note that FPR2 suppression reversed the effects of LPS on proliferation, apoptosis, invasion, migration and angiogenesis of trophoblasts. Elucidating the mechanisms of FPR2 in the process of inflammation may yield new disease markers and therapeutic targets for PE.

## Data Availability

All data generated during this study are included in this published article.
